# Meaning-centered psychotherapy for Chinese caregivers of patients with advanced cancer: Cultural and linguistic adaptation

**DOI:** 10.1007/s00520-026-10938-x

**Published:** 2026-07-02

**Authors:** Naomi Takemura, Arthur Cheuk-Man Li, Allison J. Applebaum

**Affiliations:** 1 School of Nursing, The Hong Kong Polytechnic University, Hung Hom, Hong Kong, SAR China; 2 JC STEM Lab of Digital Oncology Care Enhancement, Hong Kong, SAR China; 3 LKS Faculty of Medicine, The University of Hong Kong, Hong Kong, SAR China; 4 Steven S. Elbaum Family Center for Caregiving, Brookdale Department of Geriatrics and Palliative Medicine, Icahn School of Medicine at Mount Sinai, New York, NY USA

**Keywords:** Advanced cancer, Caregivers, Chinese, Meaning-centered, Cultural adaptation

## Abstract

**Purpose:**

Cancer incidence is rising in Hong Kong, with over half of common cancers diagnosed at advanced stages, placing substantial demands on families and driving high caregiver distress. Meaning-centered psychotherapy for cancer caregivers (MCP-C) shows promise in Western settings but requires cultural tailoring for Chinese caregivers whose values are shaped by Confucian ethics. This study aimed to culturally and linguistically adapt MCP-C for Chinese caregivers of patients with advanced cancer in Hong Kong to enhance acceptability, relevance and conceptual equivalence.

**Methods:**

Guided by the ORBIT model and Bernal’s ecological validity model (EVM), we conducted semistructured interviews with nine cancer caregivers and eight healthcare professionals (oncologists, nurses, social worker and clinical psychologist). The manual was translated and transcreated into traditional Chinese. Analysis used a hybrid deductive–inductive approach anchored to EVM domains with dual independent coding and consensus adjudication.

**Results:**

Adaptations spanned all eight EVM domains (language, persons, metaphors/stories, content, goals, methods, concepts and context), including reframing “meaning” around family roles and filial responsibility, integrating Cantonese idioms, simplifying terminology, rebranding as a “meaning-centered well-being program,” and enabling online delivery to improve feasibility.

**Conclusion:**

This systematic adaptation preserved therapeutic fidelity while enhancing cultural fit for Chinese caregivers. A mixed-methods feasibility study will evaluate acceptability, appropriateness and preliminary outcomes (meaning in life, distress, spiritual well-being and benefit finding) to inform subsequent efficacy trials.

**Supplementary Information:**

The online version contains supplementary material available at 10.1007/s00520-026-10938-x.

## Background

Cancer incidence continues to rise globally and within specific regions, including Hong Kong [1]. In Hong Kong, more than half of cases in the three most common cancers are diagnosed at an advanced stage [1]. While advances in cancer treatment prolong survival for patients with advanced disease, the substantial responsibility for care provision increasingly falls upon families and caregivers outside formal healthcare settings [2, 3]. Caregivers for patients with advanced cancer shoulder tremendous responsibilities and face significant challenges, including disrupted schedules, limited career advancement and social isolation [4–6]. These demands contribute to significant caregiver burden and psychological distress, with common manifestations such as burnout and anticipatory grief related to the poor prognosis of the care recipients [4–6]. This leads to clinically significant distress among caregivers, with rates ranging from 55 to 90% throughout the course of the disease [7–9]. This distress extends beyond the diagnostic phase, persisting over time and often manifesting as heightened death anxiety and unique existential concerns, including guilt, anticipatory grief [10] and despair regarding life after the patient’s death [11]. Compounding this burden, caregivers frequently feel pressured to maintain hope and a positive attitude, inhibiting open discussion of their distress [12]. Thus, caregivers of advanced cancer patients represent a highly vulnerable population requiring dedicated support.

A promising intervention is meaning-centered psychotherapy for cancer caregivers (MCP-C). Developed within a Western context, MCP-C is a logotherapeutic-oriented approach designed to help caregivers connect or reconnect to various sources of meaning [13, 14]. It has demonstrated feasibility, acceptability and promise in enhancing personal meaning, benefit finding, spiritual wellbeing and reduction of depressive symptomatology among Western caregivers of patients with glioblastoma multiforme [15, 16]. However, the applicability of MCP-C to non-Western populations remains unexplored.

To date, cultural and linguistic adaptations of meaning-centered psychotherapy have primarily focused on patient populations, most notably MCP-Ch for Chinese American patients with advanced cancer [17]. However, no published adaptation of MCP specifically for caregivers exists in a Chinese context. This gap is significant, as caregivers face unique existential challenges—including anticipatory grief, guilt and despair regarding life after the patient’s death—that are distinct from patients’ experiences [18, 19]. MCP-C was specifically developed to address these caregiver-specific sources of meaning, such as the historical legacy of the caregiving relationship and attitudinal responses to caregiving constraints [14]. While MCP-Ch has demonstrated acceptability for Chinese patients, caregivers represent a distinct population with differing needs and meaning-making processes, particularly in Confucian-influenced societies where filial piety and relational harmony profoundly shape caregiving [20]. Thus, adapting MCP-C for Chinese caregivers addresses a critical gap in psychosocial oncology care.

Crucially, the construction of meaning, identification of vital goals, sense of responsibility, comprehension of sources of meaning sources and acceptance of life circumstances are profoundly shaped by cultural, social and spiritual/religious factors [21]. MCP-C was developed within a Western sociocultural context where conceptualisations of “meaning” and “caregiving” may differ significantly from those prevalent in Chinese societies guided by Confucian values. Core Western constructs, such as individual legacy and active meaning creation, may conflict with distinctly Confucian principles emphasising filial piety, obligation and relational harmony [20]. Linguistic and conceptual equivalence is thus essential to ensure exercises resonate with Chinese caregivers’ lived experiences. Cultural adaptation is essential to maximise an intervention’s acceptability and relevance by meticulously considering the target population’s cultural values, beliefs and context [22]. Furthermore, evidence demonstrates that culturally appropriate, tailored interventions enhance receptivity to health information and programmes, ultimately improving effectiveness and outcomes [23, 24]. Therefore, this stud y aims to undertake the cultural and linguistic adaptation of MCP-C for Chinese caregivers of patients with advanced cancer, ensuring conceptual equivalence in translation and contextual relevance.

## Method

### Cultural adaptation framework

The ecological validity model (EVM) developed by Bernal et al. (1995) served as the primary framework for cultural adaptation, being the first model to detail evidence-based treatment adaptation [22]. EVM outlines eight dimensions for modifying psychological interventions: (1) language (i.e., translation should be culturally syntonic); (2) persons (i.e., the role of ethnic/racial similarities and differences in shaping the patient–therapist relationship); (3) metaphors (i.e., sayings or stories utilised in the intervention should be culturally relevant); (4) content (i.e., how to handle cultural information about values, customs and traditions in the intervention); (5) concepts (i.e., concepts or theoretical model used in the intervention); (6) goals (i.e., supportive of adaptive values from the patients’ culture); (7) methods (i.e., procedures utilised in the intervention should be culturally appropriate) and (8) context (i.e., the intervention should include broader social, economic and political contexts).

### The intervention: meaning-centered psychotherapy for cancer caregivers (MCP-C)

MCP-C is a logotherapeutic-oriented approach rooted in Viktor Frankl’s principles, encompassing the will to meaning, meaning in life, responsibility, freedom, finding meaning through courage and commitment, living one’s legacy and finding a sense of meaning [25]. Its adaptation for caregivers was developed to help them connect or reconnect to various sources of meaning [13, 14]. MCP-C specifically addresses four sources of meaning in life: The historical source, derived through reflecting on life lived in context, stemming from an unchosen past that influences present choices to replicate enjoyed legacies and avoid painful ones, thereby creating a “living legacy” impacting the future legacy given to others either directly (e.g., teaching life lessons) or by being witnessed (e.g., as a caregiver). The attitudinal source, arising from the chosen attitude toward life’s limitations, encompassing both role constraints and the emotional suffering inherent in end-of-life accompaniment within caregiving. The creative source, derived from how life is created through responsibility, self-expression in work, relationships and devoted values, alongside self-care, courageous actions and commitments. The experiential source, derived through receiving life and connecting via the five senses, and love, beauty and humor, fostering an expanded sense of connectedness. MCP-C is delivered individually across seven sessions. The initial two sessions introduce the concept of sources of meaning and identity in the context of caregiving. The subsequent four sessions each focus in depth on one of the four meaning sources. The final session facilitates reflection on future goals, including preparation for loss and envisioning new life possibilities. Didactic elements and experiential exercises are integrated throughout each session.

### Study design

The ORBIT model for behavioural treatment development guided the refinement, optimisation and future efficacy testing of MCP-C [26]. This framework delineates adaptive, iterative guidelines for early-phase intervention development through four sequential stages: Phase Ia defines intervention components through formative research; Phase Ib refines the intervention for strength and efficiency, including tailoring for special populations; Phase IIa conducts proof-of-concept trials using quasi-experimental, treatment-only designs to detect clinically significant signals and Phase IIb implements pilot testing with randomised designs to assess feasibility prior to Phase III efficacy trials. This article reports exclusively on Phases Ia and Ib. Phase Ia involved formative research to identify necessary cultural and linguistic modifications to the MCP-C manual. This was achieved through individual semi-structured interviews with two stakeholder groups: (1) healthcare professionals with Chinese cultural backgrounds and experience caring for patients with advanced cancer, who reviewed the manual and provided feedback on cultural relevance and conceptual equivalence and (2) caregivers of patients with advanced cancer, who offered insights into their lived experiences, cultural values and perceptions of the intervention content. These stakeholder inputs were systematically analysed to identify culturally inappropriate content, develop culturally relevant alternatives and determine content gaps requiring supplementation. Subsequently, Phase Ib—informed by Phase Ia findings and guided by the EVM for cultural adaptation [22]—encompassed translating and transcreating the intervention manual into Traditional Chinese. Specifically, the Phase Ia feedback directly guided the translation process by identifying concepts requiring linguistic and conceptual modification, providing culturally syntonic metaphors and examples and highlighting areas where additional culturally relevant content was needed. This process extended beyond direct linguistic translation to incorporate culturally relevant terminology and content, thereby addressing population-specific information needs [27]. Ethics approval was obtained from the respective institution.

### Participants and setting

All participants were residents of Hong Kong. Healthcare professionals were eligible if they i) were aged ≥ 18 years; ii) possessed familiarity with Chinese and Hong Kong culture and iii) had experiences caring for patients with advanced cancer. Caregivers were eligible if they i) were aged ≥ 18 years; ii) were current caregiver to a patient diagnosed of stage III or IV cancer; iii) able to read or understand Chinese and iv) scored at least 4 on the distress thermometer (DT) with distress related to caregiving. Participants were excluded for psychopathology or cognitive impairment likely to impede participation.

Healthcare professionals—including oncologist, nurse, clinical psychologist and medical social worker—were recruited through professional networks and snowball sampling through professional contacts. Caregivers were recruited through Hong Kong online platforms and cancer support groups. Written informed consent was obtained from eligible participants prior to study commencement. The present qualitative research adopted an inductive approach without a predetermined sample size. The final sample size was determined based on the information redundancy.

### Data collection

The MCP-C manual was initially translated into Traditional Chinese by a bilingual postgraduate psychology student with subject area expertise, guided by a bilingual nurse academic with extensive experience with Chinese cancer patients and caregiver contexts. A second translator, a bilingual clinical psychologist specialising in the existential–phenomenological approach, reviewed the initial draft, focusing on language accuracy and consistency with the philosophical and psychological concepts of logotherapy and MCP.

Individual semistructured interviews were conducted in Cantonese, the most common Chinese dialect in Hong Kong and the native language of all participants and the first author, by the first author (NT), a registered nurse and nurse scientist with seven years' research experience with patients with advanced cancer and their caregivers. This approach ensured methodological consistency in data collection and interpretation throughout the study period. The interview guide was developed based on the eight EVM domains [22] (Appendix 1) and explored participants’ perspectives on the cultural appropriateness of the MCP-C manual, including language clarity, conceptual relevance and suggestions for modification. Participants were provided with the MCP-C materials approximately 30 min prior to the interview to allow them to familiarise themselves with the content before providing feedback. For healthcare professionals, a summary of the MCP-C session structure and key concepts was also presented during the interview to facilitate informed feedback. Participants did not receive compensation for their time, as participation was voluntary. Sociodemographic characteristics, including age, sex, education and marital status, were also recorded.

### Data processing and analysis

Qualitative interview data were analysed using a hybrid deductive–inductive approach aligned with the EVM framework [28]. Deductive content analysis, as a theory-driven top-down process, was employed alongside inductive analysis, which adopts a bottom-up, data-driven approach [29]. Specifically, eight predefined EVM domains (i.e., language, persons, metaphors/stories, methods, content, goals, and concepts) served as the initial coding framework. Interview content was systematically coded according to these domains, after which inductive themes were developed to provide granular detail. These emergent themes were subsequently organised as subcodes within the corresponding EVM domains. All interviews were conducted and audio-recorded in Cantonese. The audio recordings were transcribed verbatim in Cantonese (traditional Chinese characters). Coding was conducted in the original Cantonese language to preserve the richness and nuance of participants’ expressions. Only after the coding process was complete were illustrative quotes translated into English for presentation in the manuscript. The translation was performed by the first author (NT), a native Cantonese speaker and bilingual researcher, and verified by a second bilingual team member to ensure accuracy and conceptual equivalence. Two researchers independently coded identical interview transcripts. Intercoder reliability was established through iterative consensus-building discussions, whereby coding divergences were systematically examined until unanimous agreement was reached on all applied codes.

### Reflexivity

The research team comprised individuals with diverse disciplinary and cultural backgrounds. The first author (NT) is a registered nurse and nurse scientist with over seven years of research experience with patients with advanced cancer and their caregivers in Hong Kong. As a native Cantonese speaker and member of the local community, NT brought cultural insider knowledge that facilitated rapport-building and nuanced interpretation of participant narratives. However, this insider status also carried the potential for assumptions based on shared cultural familiarity, which was addressed through regular team debriefing and peer review. The team included a bilingual clinical psychologist specialising in existential-phenomenological approaches and a qualitative researcher with psychology background. To enhance trustworthiness, we employed independent coding with consensus discussions, peer debriefing and reflexive journaling throughout the research process.

## Results

### Participants’ characteristics

Table 1 details the demographic characteristics of caregivers and healthcare professionals, respectively. A total of nine caregivers were recruited, with a mean age of 54.9 (10.4) years (range 36–68). The majority were female (55.6%, *n* = 5) and married (66.7%, *n* = 6), with most having completed at least secondary education (77.8%, *n* = 7). Slightly more than half of were spouses of care recipients (55.6%, *n* = 5), while the remainder were adult children (44.4%, *n* = 4). Caregivers reported moderate distress levels (the mean of DT was 4.9 (1.3)). Care recipients were patients with advanced cancers of various types, including lung (44.4%, *n* = 4), colorectal (33.3%, *n* = 3), nasopharyngeal (11.1%, *n* = 1) and prostate (11.1%, *n* = 1) cancers. The disease stage was predominantly stage IV (66.7%), with the remaining stage III (33.3%).

**Table 1:** Demographic characteristics of participants (*n* = 17)

Characteristics	Caregivers (*n* = 9)	Healthcare professionals (*n* = 8)
Gender, *n* (%)		
Male	4 (44.4%)	3 (37.5%)
Female	5 (55.6%)	5 (62.5%)
Age		
Mean (*SD*)	54.9 (10.4)	43.25 (6.54)
Relationship status, *n* (%)		
Single	3 (33.3%)	1 (12.5%)
Married/partnered	6 (66.7%)	7 (87.5%)
Education, *n* (%)		
Junior high school or below	2 (22.2%)	0 (0%)
High school	4 (44.4%)	0 (0%)
College or above	3 (33.3%)	8 (100%)
Employment status, *n* (%)		
Unemployed	1 (11.1%)	0 (0%)
Not employed—retired	2 (22.2%)	0 (0%)
Homemaker	3 (33.3%)	0 (0%)
Paid part-time employment	1 (11.1%)	0 (0%)
Paid full-time employment	2 (22.2%)	8 (100%)
Relationship to the patient, *n* (%)		
Wife	3 (33.3%)	-
Husband	2 (22.2%)	-
Daughter	2 (22.2%)	-
Son	2 (22.2%)	-
Diagnosis of patients		
Lung cancer	4 (44.4%)	-
Colorectal cancer	3 (33.3%)	-
Nasopharyngeal cancer	1 (11.1%)	-
Prostate cancer	1 (11.1%)	-
Disease stage of patients		
Stage III	3 (33.3%)	-
Stage IV	6 (66.7%)	-
Distress level (measured by Distress Thermometer), *mean (SD)*	4.9 (1.3)	

Eight healthcare professionals participated in the study, with a mean age of 43.25 (6.54) years (range 33–53). Among them, 62.5% were female, all had tertiary education, and 87.5% were married. Their professional roles included oncologist (37.5%, *n* = 3), oncology/palliative care nurses (37.5%, *n* = 3), a medical social worker (12.5%, *n* = 1) and a clinical psychologist (12.5%, *n* = 1).

### EVM domains

Table 2 summarises the session-by-session adaptations to MCP-C, specifying the adaptation type (e.g., content addition/refinement/substitution) and the corresponding EVM domains (language, persons, metaphors, content, concepts, goals, methods, and context) to illustrate how cultural fit was operationalised across the intervention.

**Table 2:** Adaptations to MCP-C by session, adaptation type, and EVM

Session	Session title	Content	Adaptation type (content addition/refinement/substitution)	Adaptation	EVM
1	Concepts and sources of meaning	Introductions; review of concepts of meaning and caregiving, and sources of meaning; experiential exercise: meaningful moments optional reading: copies of man’s search for meaning	Content refinement Content substitution	Refined handout to simplify the definitions of different sources of meaning Therapists will tell and capture the main points of Frankl’s story verbally	Language Methods/strategies, Metaphors
2	Cancer caregiving and meaning: identity before and after becoming cancer caregiver	Discussion of sense of identity before and after becoming a cancer caregiving; experiential exercise: who am I? Homework reflection: session 3 experiential exercise	Content refinement Content addition Content addition	Handout used for the discussion of identity before and after becoming a cancer caregiver was refined to show the relationship of cancer patient and caregiver, and also the potential changes that led to suffering In defining ‘identity,’ examples were added, including ‘family,’ ‘roles,’ ‘duties and responsibilities,’ ‘beliefs,’ and ‘values’ In defining ‘accomplishment,’ emphasis was added on ‘dream,’ ‘achievements,’ and ‘things you are good at’	Content, concepts Concepts Concepts
3	Historical source of meaning: life as a living legacy	Discussion of life as a legacy that has been given (past), one lives (present), and gives (future); experiential exercise: historical sources of meaning-past, present, and future experiential exercise; homework reflection: session 4 experiential exercise and optional sharing of one’s story	Content refinement Content refinement	Experiential exercise was refined where the present was integrated with the discussion of past and future meaning for simplification and emphasis on connectedness Used cultural idiom, “passing the torch” (薪火相傳) to convey Confucian value of continuity	Concepts Metaphors
4	Attitudinal sources of meaning: encountering life’s limitations	Discussion of confronting limitations and challenges associated with caregiving; experiential exercise: encountering life’s limitations experiential exercise; introduction to legacy project; homework reflection on session 5 experiential exercise	Content removal/refinement Content refinement Content addition	The question “what would your loved one with cancer consider to be a good death?” was made optional depending on the situation of the patient’s prognosis Used cultural idiom “life begets life without end” (生生不息) to convey the enduring and expansive nature of legacy Concrete examples were given for the Legacy Project	Goals Metaphors Content
5	Creative source of meaning: engaging in life fully	Discussion of creativity, courage and responsibility; experiential exercise: creative sources of meaning homework reflection: session 6 experiential exercise	Content addition	Emphasis was added on ‘giving to the world’ when defining the concept of creative source of meaning	Concepts
6	Experiential sources of meaning: connecting with life	Discussion of experiential sources of meaning, such as love, nature, art, and humor; experiential exercise: love, beauty, & humor homework: planning/completion of legacy project and self-care project for presentation in Session 7	Content addition Content refinement	Emphasis was added on ‘receiving from the world’ when defining the concept of experiential source of meaning Reframed the concept of humor as “self-mockery” (自嘲), “mutual teasing” (互嘲), and “finding joy in adversity” (苦中作樂)	Concepts Concepts
7	Transitions: reflections, and hopes for the future	Review of sources of meaning, reflections on lessons learned; experiential exercise: hopes for the future experiential exercise; goodbyes	N/A	N/A	
All sessions			Content refinement Content refinement	Simplified manual language to fit the general public renamed meaning-centered psychotherapy to “meaning-centered well-being program” to counter mental health stigma	Context Methods/strategies, context

#### Language

Caregivers articulated discomfort with Western clinical terminology, emphasising that direct translations failed to capture embodied emotional experiences. One caregiver mentioned: “When I hear the word ‘meaning,’ it sounds like something grand and philosophical—something I don’t have time to think about. I just focus on getting through each day and taking care of my husband.”

While retaining the direct translation, we explicitly reframe “meaning” as actionable human experience grounded in ‘life purpose’ and ‘what we live for.’ This anchors the concept in tangible caregiving roles rather than abstract ideals. Furthermore, translating ‘meaning-making’ as ‘尋找意義’ (search for meaning) appeared to be a philosophical puzzle. Another caregiver explained: “I don’t think I'm ‘searching for meaning.’ It’s more like I find purpose in the small moments—when my mother smiles, when I know I've done my duty.”

In response, we incorporated this language into the manual, describing meaning as emerging through “small moments of connection, duty fulfilment and reframed perspectives on daily tasks.”

#### Persons

In this Hong Kong-based study, participants were recruited exclusively from local public hospitals, while therapists were native Cantonese speakers born and raised in Hong Kong. Though ethnic concordance was present, cultural competence proved more consequential than shared ethnicity alone. One caregiver stated: “It’s not enough that the therapist speaks Cantonese. They need to understand how we think—how filial piety works in our families, why we don’t talk about death openly.”

A clinical psychologist elaborated: “Therapists need to ask, not assume. They shouldn’t treat all Chinese families the same. Hong Kong families are different—some are very traditional, others more Westernised.”

This feedback directly informed two adaptations. First, we trained therapists to probe participants’ subjective interpretations of culturally relevant concepts rather than imposing monolithic cultural narratives. Therapists now ask questions such as, “How is filial piety expressed in your family?” Second, we added guidance in the therapist manual on navigating Hong Kong’s sociocultural duality, where caregivers navigate traditional Confucian duties alongside pragmatic urban realities.

#### Metaphors

Healthcare professionals recommended incorporating culturally resonant idioms to make abstract concepts resonate with the target population’s lived experience, thereby deepening comprehension and cultural relatability. One medical social worker suggested: “We have so many rich idioms in Cantonese that capture these ideas perfectly. ‘Passing the Torch’ (薪火相傳) would work well for intergenerational responsibility.”

A caregiver also affirmed the value of familiar expressions: “When you use sayings we grew up with, it feels like you understand us. It’s not foreign or clinical—it’s part of our lives.”

We strategically integrate local idioms to activate cultural schemas and values. By embedding metaphors drawn from shared linguistic heritage, the manual transforms theoretical constructs into culturally intuitive narratives. The idiom “Passing the Torch” (薪火相傳) articulates Confucian continuity in the Historical Sources of Meaning session, evoking intergenerational responsibility and cultural perpetuity. Similarly, “Life Begets Life Without End” (生生不息) embodies the enduring, expansive nature of legacy in the Attitudinal Sources of Meaning session, aligning with Taoist principles of cyclical renewal. This approach transcreates—not merely translates—meaning.

#### Content

Participants highlighted the need to embed existential concepts with Confucian value, such as familial piety, relational harmony and intergenerational legacy throughout intervention materials. One caregiver explained:

“In our culture, suffering isn’t just suffering—it’s part of duty. You endure because it’s your responsibility as a child or a spouse. That’s what makes it meaningful.”

A palliative care nurse added:

“If you frame endurance as filial devotion, it resonates. If you frame it as ‘coping with distress,’ it feels like you’re pathologising something that is culturally expected.”

Abstract ideas were transformed into culturally recognizable narratives; for example, discussions of suffering reframed endurance as filial devotion. This cultural embedding manifested most concretely in the Legacy Project, where therapists dynamically introduced examples aligned with caregivers’ lived experiences and personal values. Traditional caregivers hesitant toward talent-dependent activities (e.g., photo albums or music composition) were offered culturally resonant alternatives like writing letters to their loved one (the care recipient), initiating heartfelt dialogues previously hindered by caregiving responsibilities or sharing caregiving experiences (or the patient’s story) with their children. These options prioritised Confucian ethical connections by enabling caregivers to transform experiences into intergenerational continuity or mutual understanding, while therapists emphasised client autonomy in selecting projects grounded in personal values.

#### Goals

Participants emphasised that treatment goals should be anchored in family roles and obligations rather than individual aspirations alone. One caregiver stated:

“My goal isn’t about me. It’s about being there for my husband and making sure our children are okay. That's what gives me purpose.”

A palliative care nurse confirmed:

“Caregivers don’t separate their own wellbeing from their family’s wellbeing. Goals need to reflect that interconnectedness.”

We framed treatment goals through relational lenses, emphasising filial responsibility and collective wellbeing. Additionally, the medical social worker raised a concern about a specific question in the Attitudinal Sources of Meaning session:

“Asking a caregiver ‘What would your loved one consider a good or meaningful death?’ could be distressing, especially if the patient’s prognosis is uncertain. I’d recommend making this optional.’

We adopted this recommendation, redesigning the question as optional contingent upon the patient's prognosis to honour cultural norms of hope preservation during medically uncertain trajectories.

#### Methods/strategies

Participants highlighted practical barriers to participation, including time constraints, caregiving burden and mental health stigma. One caregiver explained:

“I don’t have time to read long materials. If you can just tell me the key points during the session, that would be much easier.”

The clinical psychologist echoed:

“Many caregivers won’t attend anything labelled ‘psychotherapy’—they see it as admitting they can’t cope. You might need to rebrand it.”

We adopted two key methodological adaptations. First, therapists verbally distill the essence of Viktor Frankl’s narratives during sessions rather than assigning textual materials to reduce cognitive burden for time-constrained caregivers. Second, the program title was adapted to “meaning-centered well-being program,” replacing “psychotherapy” terminology to circumvent mental health treatment stigma prevalent in Chinese communities. Additionally, consistent with prior U.S. pilot work [15], we transitioned the intervention from in-person delivery to an online format to enhance accessibility, accommodate unpredictable caregiving schedules and eliminate travel burdens. This digital adaptation provides psychological safety through familiar, private environments for discussing existential themes.

#### Context

In Hong Kong, a relatively well-educated region, variable health literacy exists and this consequently influences engagement with interventions. An oncologist advised: “Some caregivers will struggle with philosophical or medical jargon. You need to simplify the language without losing the depth of the concepts.”

One caregiver confirmed this concern:

“Some of the words feel too academic. I don’t have time to figure out what 'existential despair' means—I just know I feel hopeless sometimes.”

Based on this feedback, we systematically simplified medical and philosophical terminology throughout the manual. For example, “existential despair” was translated as “profound hopelessness” and “transcendence” as “finding purpose beyond suffering.” These changes enhanced readability while preserving conceptual depth.

#### Concepts

Healthcare professionals recommended reinterpreting Frankl’s existential concepts through a Confucian relational lens. The clinical psychologist explained:

“The idea of ‘freedom of will’ sounds very individualistic. In our culture, freedom isn’t about doing what you want—it’s about making purposeful choices within your responsibilities, your role. The Confucian concept of ‘settle down and get on with one’s pursuit’ (安身立命) captures this better.”

A caregiver added:

“I don’t think of myself as ‘creating meaning.’ I think of myself as giving to my family—that’s where meaning comes from.”

We reinterpreted “freedom of will” through the Confucian concept of 安身立命, emphasising how individuals exercise agency to make purposeful choices and establish their moral footing within constrained circumstances. Creative values were reframed as “giving to the world” and experiential values as “receiving from the world,” translating individualistic constructs into interdependent exchanges that honour the Confucian understanding of life as a web of mutual obligations. Additionally, we expanded “accomplishment” to encompass culturally significant markers like fulfilled dreams and recognised achievements, and redefined humour through culturally resonant expressions like self-mockery (自嘲), mutual teasing (互嘲) and finding joy in adversity (苦中作樂).

## Discussion

The study applied the EVM and the ORBIT model for behavioral treatment development to culturally and linguistically adapt MCP-C for Chinese caregivers of patients with advanced cancer. Our goal was to enhance acceptability, relevance, comprehensibility and completeness for this specific population. To our knowledge, this is the first cultural adaptation of a meaning-centered psychotherapy for cancer caregivers tailored to Chinese caregivers of individuals with advanced cancer.

A structured framework and rigorous process are essential for cultural adaptation. The EVM [22] enabled us to go beyond translation and systematically identify core cultural values and contextual factors—including filial piety, family harmony, role obligations, respect for authority, stigma and “face” preservation and preferences for indirect communication—which were embedded throughout session content, exercises and examples. Integrating such culturally sensitive elements into psychosocial interventions is known to enhance their impact [30]. Guided by the ORBIT model and the eight EVM domains (language, persons, metaphors, content, concepts, goals, methods and context), our adaptation proceeded in a stepwise manner and drew on meaningful input from both intervention beneficiaries—Chinese caregivers of patients with advanced cancer—and healthcare professionals.

Our findings align with prior cultural adaptations of MCP for Chinese populations. The MCP-Ch adaptation for Chinese American patients with advanced cancer similarly employed the EVM to guide cultural and linguistic modifications. Adaptations included shortening the number of sessions, using the teach-back method to clarify abstract concepts and incorporating culturally relevant resources [17, 31]. Both adaptations identified comparable cultural considerations: the need for simplified language to convey existential concepts, the importance of culturally resonant metaphors and the integration of Confucian values such as filial piety and family harmony. However, our adaptation addresses a distinct population—caregivers rather than patients—and specifically targets Hong Kong Chinese caregivers, who navigate a unique socio-cultural context characterised by traditional Confucian values alongside pragmatic urban realities. Recent refinement work on MCP-Ch further supports our approach. Stakeholder feedback highlighted the need for increased protocol flexibility, simplified content and additional provider training to enhance acceptability and implementation in real-world settings [32]. These recommendations parallel our findings, particularly the importance of adapting delivery methods to reduce caregiver burden (e.g., online delivery and verbal distillation of materials) and the need for therapists to possess cultural competence beyond shared ethnicity. Our study extends this literature by demonstrating how cultural adaptation frameworks can be applied to caregiver populations, a group often overlooked in psychosocial oncology research.

A successful adaptation necessitates contributions from both healthcare professionals and beneficiaries [33]. In our key informant interviews, healthcare professionals prioritised clinical fidelity, safety and cultural alignment based on their experience with cancer caregivers, while potential beneficiaries highlighted preferences for tone, examples and delivery that resonated with their lived realities. This complementary perspective was critical: healthcare professionals helped safeguard therapeutic integrity and feasibility in oncology settings, whereas caregivers prompted refinements to language, metaphors and activities to ensure emotional resonance and practicality under high caregiving burden.

Regarding participant characteristics, our caregiver sample primarily comprised spouses and adult children supporting patients with advanced disease, well-aligned with the target population. However, the substantial burden of caregiving likely limited participation among those experiencing acute distress, potentially biasing preferences toward less intensive formats. We actively mitigated this limitation by designing scalable session elements and emphasising relatable themes. Future adaptations should prioritise recruitment strategies that engage higher-distress caregivers to comprehensively assess intervention tolerability.

Culturally adapted interventions require rigorous evaluation, as well-adapted therapies may enhance treatment outcomes [34]. Therefore, evaluating intervention effectiveness is essential not only to assess clinical impact but also to determine the sufficiency of the cultural adaptation. Building on this adapataion, we will conduct a feasbility study using a mixed-methods design to examine the acceptability, appropriateness and feasibility of culturally adapted MCP-C among Chinese caregivers of patients with advanced cancer. Quantitative measures will include feasibility metrics and clinical outcomes such as meaning in life, psychological distress, spiritual well-being and benefit finding. The qualitative component will explore caregivers' perceptions and satisfaction with the intervention, examine their perceived treatment effects and identify factors influencing engagement with MCP-C. This step is vital to determine whether further modifications are needed before testing the efficacy of the culturally adapted intervention in a full-scale randomised controlled trial.

## Limitations

Several limitations warrant acknowledgment. First, caregivers with severe distress were underrepresented in our adaptation sample, potentially limiting generalizability to higher-need subgroups. Nevertheless, participants provided valuable insights reflective of diverse caregiving experiences. Second, while the original MCP-C developer was not part of the initial cultural adaptation team, her expertise was consulted during the adaptation process described in this manuscript and subsequently during the feasibility testing phase of the broader research programme [35] to ensure therapeutic integrity of the modifications. This feasibility testing was conducted after the adaptation process reported here was completed.

## Conclusion

In summary, using the EVM and ORBIT frameworks, we systematically adapted MCP-C for Chinese caregivers of patients with advanced cancer; integrating culturally congruent language, metaphors and practices and pragmatically tailoring methods to caregiving constraints. These modifications, co-informed by healthcare professionals and caregivers, aim to preserve clinical fidelity while enhancing cultural and contextual fit. Next steps include feasibility testing and rigorous efficacy trials to establish the impact of culturally adapted MCP-C on caregiver well-being in advanced cancer care.

## Supplementary Information

Below is the link to the electronic supplementary material.

ESM 1 (DOCX 19.7 KB)

## Data Availability

The data that support the findings of this article are not publicly available but can be requested from the corresponding author.
