# Supplementary material for: Meaning-centered psychotherapy for Chinese caregivers of patients with advanced cancer: Cultural and linguistic adaptation
Source: Support Care Cancer. 2026 Jul 2;34(7):721. doi: 10.1007/s00520-026-10938-x (PMC13328167; doi:10.1007/s00520-026-10938-x)
Supplement: Supplementary file 1 — (DOCX 19.7 KB) [file 520_2026_10938_MOESM1_ESM.docx]

**Appendix 1. Semi-Structured Interview Guide**

1. **Language**

**Healthcare professionals:**

1. How would you explain "meaning" to Hong Kong caregivers without it sounding like a grand, abstract idea?
2. How would you translate "meaning-making" into Cantonese in a way that feels relatable to daily caregiving?
3. Are there Cantonese terms that better capture "purpose emerging through small moments of connection, duty fulfilment, and reframed perspectives"?

**Caregivers:**

1. In your own words, what does "meaning" mean to you in your caregiving role?
2. What words feel most natural and comfortable when discussing difficult topics like death and dying?
3. Are there any words or phrases that feel confusing or too abstract?
4. **Persons**

**Healthcare professionals:**

1. What characteristics should a therapist have to work effectively with Hong Kong caregivers?
2. How should therapists approach culturally sensitive topics in this context?
3. How can therapists build trust and rapport with caregivers from this cultural background?

**Caregivers:**

1. What would you look for in a therapist for a program like this?
2. What would help you feel comfortable discussing personal concerns with a therapist?
3. Are there any cultural factors that would affect your relationship with a therapist?
4. **Metaphors**

**Healthcare professionals:**

1. What culturally relevant stories, idioms, or metaphors could help illustrate the intervention's concepts?
2. Are there any metaphors or examples that might not translate well across cultures?

**Caregivers:**

1. Are there any stories, examples, or sayings in the materials that feel familiar or unfamiliar to you?
2. What proverbs or sayings resonate with your caregiving experience?
3. **Content**

**Healthcare professionals:**

1. What aspects of the intervention content might need modification for Hong Kong caregivers?
2. What topics or exercises might be culturally sensitive and require adjustment?
3. Are there culturally specific sources of caregiver distress that the intervention should address?

**Caregivers:**

1. What aspects of the intervention content feel most relevant to your experience?
2. Are there topics or exercises that you would find uncomfortable or inappropriate?
3. What cultural beliefs or practices help you cope with caregiving challenges?
4. **Goals**

**Healthcare professionals:**

1. What should be the primary goals of this intervention for Hong Kong caregivers?
2. How should treatment goals balance individual needs with family obligations?
3. What considerations should guide goal-setting for caregivers in this cultural context?

**Caregivers:**

1. What would you hope to gain from a program like this?
2. What goals feel most important to you as a caregiver?
3. **Methods/ Strategies**

**Healthcare professionals:**

1. What delivery format (e.g., in-person, online, group, individual) would work best for Hong Kong caregivers?
2. Are there any practical barriers to participation that should be addressed?
3. What session structure (e.g., length, frequency, number of sessions) would be most appropriate?

**Caregivers:**

1. Would you prefer in-person or online sessions? Why?
2. How long should each session be, and how many sessions would feel manageable?
3. What would make it easier for you to attend sessions?
4. **Context**

**Healthcare professionals:**

1. What social, economic, or systemic factors affect caregiving in Hong Kong?
2. How might health literacy influence engagement with this intervention?
3. What practical considerations should be taken into account for Hong Kong caregivers?

**Caregivers:**

1. What challenges do you face as a caregiver in Hong Kong?
2. What support would be most helpful to you?
3. What practical barriers might prevent you from participating in a program like this?
4. **Concepts**

**Healthcare professionals:**

1. How do Frankl's existential concepts resonate with Chinese cultural values?
2. Are there any theoretical concepts that might need reinterpretation for this population?
3. How might caregivers in Hong Kong understand concepts like freedom, responsibility, and purpose in the context of caregiving?

**Caregivers:**

1. How do you understand the idea of finding meaning through difficult experiences?
2. What does "freedom" mean to you in the context of caregiving?
3. How does your cultural or religious background shape your understanding of life, death, and purpose?

**Overall recommendations:**

**All Participants:**

1. What modifications would make this intervention more relevant and acceptable to Hong Kong caregivers?
2. What should be added, removed, or changed?
3. Do you have any other recommendations for adapting this intervention?
